# Effect of Diazepam Sedation on Arterial Oxygen Saturation and Patient Tolerance During Esophagogastroduodenoscopy

**DOI:** 10.1155/DTE.3.171

**Published:** 1997

**Authors:** G. M. Malik, M. Mubarik, S. A. Kadla, J. A. Basu, H. Tajamul

**Affiliations:** 1 Department of Medicine Government S.M.H.S. Hospital Medical College Srinagar Kashmir 190 010 India; 2 Nalbandpora Safakadal Srinagar Kashmir 190 002 India

## Abstract

The impact of intravenous (I/V) diazepam sedation on arterial oxygen saturation (SaO_2_) and patient tolerance was studied in 100 patients in the age group of (27–60) years, who were subjected to esophagogastroduodenoscopy (EGD) for different indications. Equal number of patients in the same age group, who did not receive the sedation acted as controls. It was observed that SaO_2_ showed a transient fall during the first few minutes of the endoscopic procedure even without sedation and diazepam administration did not significantly (p > 0.05) aggravate this oxygen desaturation. It was also observed that diazepam sedation increased the procedure tolerance of the patients when compared with controls (p < 0.01). In conclusion, it was inferred that I/V diazepam may act as a safe and favourable sedative for EGD.


*Diagnostic and Therapeutic Endoscopy*, 1997, Vol. 3, pp. 171–175
Reprints available directly from the publisher
Photocopying permitted by license only

© 1997 OPA (Overseas Publishers Association)
Amsterdam B.V. Published in The Netherlands
by Harwood Academic Publisher
Printed in Singapore

### Effect of Diazepam Sedation on Arterial Oxygen Saturation and Patient Tolerance During Esophagogastroduodenoscopy

G. M. MALIK, M. MUBARIK, S. A. KADLA, J. A. BASU and H. TAJAMUL

*Department of Medicine, Government S.M.H.S. Hospital, Medical College, Srinagar — 190 010, Kashmir (India)*

*(Received 27 May 1996; in final form 2 September 1996)*

**The impact of intravenous (I/V) diazepam sedation on arterial oxygen saturation ($SaO_2$) and patient tolerance was studied in 100 patients in the age group of (27–60) years, who were subjected to esophagogastroduodenoscopy (EGD) for different indications. Equal number of patients in the same age group, who did not receive the sedation acted as controls. It was observed that $SaO_2$ showed a transient fall during the first few minutes of the endoscopic procedure even without sedation and diazepam administration did not significantly ($p > 0.05$) aggravate this oxygen desaturation. It was also observed that diazepam sedation increased the procedure tolerance of the patients when compared with controls ($p < 0.01$). In conclusion, it was inferred that I/V diazepam may act as a safe and favourable sedative for EGD.**

*Keywords:* Diazepam, sedation, esophagogastroduodenoscopy, arterial oxygen saturation, tolerance

### INTRODUCTION

Esophagogastroduodenoscopy (EGD) is associated with low morbidity and mortality and is considered a safe procedure even in critically ill patients in experienced hands. Although considered safe, there is confusion surrounding what is appropriate monitoring for patients who receive conscious sedation during EGD. One potential complication during EGD is arterial oxygen desaturation, which may be harmful or even detrimental to patients of cardiopulmonary disease [1,2]. Despite the increase in patient tolerance during EGD, the time course effects of sedation on arterial oxygen saturation in patients having, otherwise, no contraindication to the procedure or sedation have not been well analyzed [3]. This study was, thus, designed to see

Address for correspondence: Dr. Mohammad Mubarik Naqash, C/O., Nalbandpora Safakadal, Srinagar — 190 002, Kashmir (India).

the effect of intravenous diazepam sedation on endoscopy induced arterial oxygen desaturation and also to determine its effects on procedure tolerance of the patients.

### MATERIAL AND METHODS

### Patients and Controls

Esophagogastroduodenoscopy was performed on 1723 patients for various indications from December 1992 to September 1995. The present study was prospective and non-randomized, conducted on 200 patients selected from among the above total, out of whom 100 patients in the age group of 27–60 (37 ± 8.55) years acted as the study group and an equal number of the same age group (35 ± 9.75 years) as the control group (Table I).

Both the study and the control group were evaluated in detail to rule out any contraindication to the procedure. The exclusion criteria were: unstable hemodynamic status; acute hemorrhage; history of cardiovascular, respiratory or any other significant illness; any significant abnormality of the routine laboratory parameters, electrocardiogram (prior to and after the procedure) and X-ray chest (PA view).

Intravenous diazepam sedation (10 mgs) was given slowly over a period of one minute to the study group about 3 minutes prior to EGD, after ruling out any contraindication to diazepam sedation. The procedure was performed by two qualified gastroenterologists with six assistants in the endoscopy laboratory of SMHS Hospital, Srinagar (Kashmir).

### Parameters Studied

The arterial oxygen saturation was recorded at 2 minutes prior, at the start, after every 2 minutes and two minutes after the completion of procedure in both the groups, using Nelcor pulse oxymeter.

The procedure tolerance score of the patients,

TABLE I Background details, duration of procedure and diagnosis on EGD among the study and the control groups

| Patient particulars | Study group ($n$ = 100) No. of patients | Control group ($n$ = 100) No. of patients |
|---|---|---|
| 1) BACKGROUND DETAILS | | |
| i) Age (years) | | |
| 27–38 | 41 | 34 |
| 39–50 | 43 | 47 |
| 51–60 | 16 | 19 |
| ii) Sex | | |
| Males | 67 | 59 |
| Females | 33 | 41 |
| iii) Smoking history | | |
| Smokers | 21 | 14 |
| Non-smokers | 79 | 86 |
| 2) DURATION OF PROCEDURE (mins.) | 9.4 ± 0.9 | 9.8 ± 1.2 |
| 3) DIAGNOSIS ON EGD | | |
| Normal procedure | 53* | 34 |
| Benign lesions | 41 | 56 |
| Malignant lesions | 6 | 10 |

*$p < 0.05$.

as calculated, was graded as 5-Excellent (no cough, no retching), 4-Good (1 cough or retching), 3-Fair (2 coughs or retchings), 2-Poor (3 or 4 coughs or retchings) and 1-Very poor (more than 5 coughs or retchings) [3,4].

### Statistical Analysis

Statistical comparisons were based on student's 't' test. A value of 0.05 or less was considered significant.

### RESULTS

The background details of patients and mean duration of the procedure in the study group did not differ significantly from those in the control group ($p > 0.05$). However, normal procedure was observed more frequently in the study group with a significant difference when compared to controls $p < 0.05$ (Table I).

Arterial oxygen saturation ($SaO_2$) fell remarkably during the first several minutes of the procedure even without sedation. After this transient initial drop, the $SaO_2$ gradually increased and returned to the basal value even during the procedure in the control group. However, in diazepam pre-treated patients, $SaO_2$ continued to remain at lower levels till the extubation of endoscope, after which it finally increased back to the basal value. The difference in $SaO_2$ levels between the two groups was not significant at any stage of the procedure (Fig. 1).

In the control group only 5 out of 100 patients showed $SaO_2$ of <90%, with just 3 showing a saturation fall of >7%. However, in the diazepam pre-treated group, 7 out of 100 patients showed $SaO_2$ of <90%, with just 3 showing a saturation fall of >7% (*note*: more than 7% $SaO_2$ fall or $SaO_2$ of <90% is considered a risk facor for cardiac arrythmias) [4].

The tolerance score of $3 \pm 0.3$ for the study group was significantly higher as compared to $1.6 \pm 0.4$ in case of the control group $p < 0.01$ (Table II).

### DISCUSSION

Esophagogastroduodenoscopy (EGD) is reported to be a safe procedure with little morbidity and mortality especially in experienced hands [1,2]. With a significant increase in the number of such procedures being performed, even small rates of complications have now become magnified. Thus recognition of factors, that may enhance the safety of these procedures is crucial [4]. Cardiopulmonary complications account for 50% of EGD related morbidity and may be influenced by such variations as age, endoscope size and the use of systemic sedation [4,5].

The present study was undertaken to evaluate the effects of intravenous (I/V) diazepam sedation on arterial oxygen saturation ($SaO_2$) and the patient tolerance during EGD. The exclusion criteria for the selection of patients for both the study and the control groups were strictly followed to rule out any contraindication to the procedure and the diazepam sedation. Clearly, I/V diazepam sedation increased the patient tolerance in our study. This can be attributed to its sedative and anti-anxiety properties. A favourable effect on patient tolerance has also been reported earlier [3,6]. We observed that $SaO_2$ dropped just after the insertion of endoscope in both the groups. However, in the control group $SaO_2$ increased back to the basal level even during the procedure. On the other hand, sedation by diazepam prolonged the fall in $SaO_2$ until extubation, but without aggrevating the lowest values of oxygen saturation. We believe that the better patient tolerance could be at the expense of some degree of arterial oxygen desaturation depending upon various factors like prior condition of the patient, age etc.

Several previous studies have shown that hypoxia occurs during EGD, which has been attributed variously to pre-treatment, the presence of

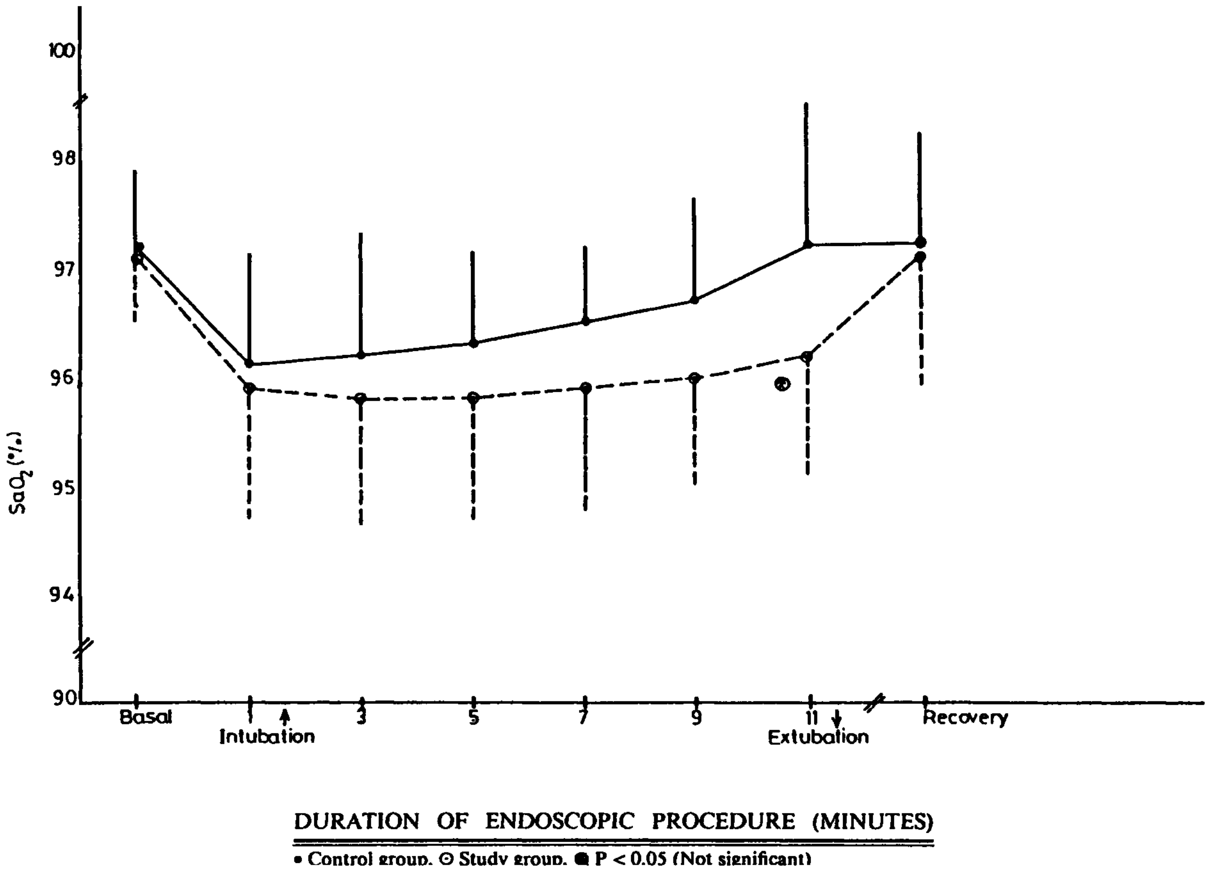

FIGURE 1 Time course changes in arterial oxygen saturation ($S_aO_2$) during the endoscopic procedure. Endoscopy induced a transient fall in $SaO_2$ in the first few minutes among both the study and the control groups. However, $SaO_2$ continued to be at a lower level till extubation in the study group (Note: vertical lines represent mean ± standard deviation).

TABLE II Showing comparison of the procedure tolerance score between the study and the control groups

| Patient group | No. of patients | Tolerance score Mean ± SD | p value |
|---|---|---|---|
| Study group (with diazepam) | 100 | 3 ± 0.3 | $p < 0.01$ (S) |
| Control group (without diazepam) | 100 | 1.6 ± 0.4 | |

SD = Standard deviation, S = Significant, Tolerance score: 5-Excellent, 4-Good, 3-Fair, 2-Poor, 1-Very poor.

endoscope in the oropharynx and aspiration of gastric contents [3,4,6–9]. However, the effect of sedation on $SaO_2$ is still much debated. Some have reported that diazepam sedation led to greater falls in saturation, particularly during the intubation procedure, [4] while as, some feel that sedation by diazepam or midazolam did not induce further desaturation over their respective control groups [3,6,7]. These conflicting observations could be due to the result of the lack of well controlled time course studies as also the wide variety of sedatives use in the endoscopic procedures [3].

The time course changes in $SaO_2$ during EGD, as observed in our study, demonstrated that even without sedation, there occurred a transient drop in $SaO_2$. After the initial desaturation, $SaO_2$ rose and returned back to its basal level despite the presence of endoscope in the oropharynx. With sedation by diazepam, $SaO_2$ showed the same initial drop as in patients without sedation. Although $SaO_2$ stayed at a decreased level until extubation, the lowest $SaO_2$ value, as observed in the diazepam treated group, was not statistically significant when compared with the control group.

Large falls in saturation (>7%) or $SaO_2$ of <90% are reported to increase the risk of cardiac arrythmias. [4] In our control group, 5 out of 100 patients showed $SaO_2$ of <90%, with 3 showing a saturation fall of >7%. In the diazepam treated group, 7 out of 100 patients showed $SaO_2$ of <90%, with just 3 showing a saturation fall of >7%. However, none of our patients in either group developed any cardiopulmonary problem as detected clinically. Therefore, we conclude that slowly given 10 mgs of I/V diazepam does not aggravate the EGD induced desaturation and can be used as a safe sedative during the procedure with a better patient tolerance. However, any contraindication to the procedure and the sedative needs to be ruled out before performing EGD.

### *References*

[1] Dark, D. S., Donald, R. C., Wesselius, L. J. Arterial oxygen desaturation during gastrointestinal endoscopy. *Am. J. Gastroentrol.* 1990; **85**(10): 1317–1321.

[2] Fleischer, D., Monitoring the patient receiving conscious sedation for gastrointestinal endoscopy — Issues and guidelines. *Gastrointest. Endosc.* 1989; **35**: 262–266.

[3] Kinoshita, Y., Ishido, S., Nishiyama, K., *et al.* Arterial oxygen saturation, blood pressure and pulse rate during upper gastrointestinal endoscopy — Influence of sedation and age. *J. Clin. Gastroenterol.* 1991; **13**(6): 655–660.

[4] Liberman, D. A., Wuertker, C. K., Katon R. M. Cardiopulmonary risk of esophagogastroduodenoscopy. *Gastroenterol.* 1985; **88**: 468–472.

[5] Katon, R. M. Complications of upper gastrointestinal endoscopy in gastrointestinal bleeder. *Dig. Dis. Sci.* 1981; **26**: 47S–54S.

[6] Lavies, N. G., Creasy, T., Harris, K., *et al.* Arterial oxygen saturation during upper gastrointestinal endoscopy — Influence of sedation and operator experience. *Am. J. Gastroenterol.* 1988; **83**: 618–622.

[7] Whorwell, P. I., Smith, C. L., Foster, K. I. Arterial blood gas tension during upper gastrointestinal endoscopy. *Gut* 1976; **17**: 797–800.

[8] Rozen, P., Fireman, Z., Gilet, T. Arterial oxygen tension changes in elderly patients undergoing upper gastrointestinal endoscopy. II. Influence of the narcotic premedication and endoscope diameter. *Scan. J. Gastroenterol.* 1981; **16**: 299–303.

[9] Rozen, P., Firemen, Z., Gilet, T. The causes of hypoxiemia in elderly patients during endoscopy. *Gastrointest. Endosc.* 1982; **28**: 243–246.

